# Social disparities in students’ intention to enter higher education during the COVID-19 pandemic

**DOI:** 10.1371/journal.pone.0267978

**Published:** 2022-05-04

**Authors:** Johannes Stark, Annabell Daniel, Mathias Twardawski

**Affiliations:** 1 Kuehne Logistics University, Hamburg, Germany; 2 Leibniz Institute for Research and Information in Education (DIPF), Frankfurt a. M., Germany; 3 Department of Psychology, Ludwig-Maximilians-Universität München, Munich, Germany; Eötvös Loránd University, HUNGARY

## Abstract

Research consistently shows that students from academic households are more likely to enter higher education than students from non-academic households. These inequalities are only secondarily due to differences in performance (i.e., primary effects), but mostly due to students’ decision making behavior (i.e., secondary effects). The relative share to which primary effects and secondary effects mediate the effect of students’ educational background on their intention to enter higher education is affected by external conditions. One significant external influence that may have had an impact on social disparities in students’ educational choices is the COVID-19 pandemic. Herein, we present data from *N* = 596 upper secondary students (41.6% from non-academic households) that were collected in Germany in April 2021. Building on rational choice theory, we scrutinized students’ expected benefits (i.e., employment prospects and personal significance), costs (i.e., direct costs and opportunity costs), and subjective probability of success in pursuing higher education as important psychological pillars for their intention to enter higher education. Results show that about 14% of social differences in students’ intention to enter higher education were due to primary effects, whereas almost 77% were explained by secondary effects. Specifically, we found that differences in the evaluation of benefits most strongly contributed to social inequalities in students’ intention to enroll in higher education. Compared to research on pre- COVID-19 cohorts, our results point to shifts in existing patterns of inequalities in the wake of the COVID-19 pandemic.

## Introduction

Obtaining a university degree is highly beneficial in Western societies, promising entry into the labor market at a relatively high wage level, as well as access to socially prestigious professions [1]. Startlingly, there is ample evidence that students’ higher education (HE) attainment is largely determined by their parents’ educational background. Specifically, students from academic households (i.e., at least one of their parents holds a HE degree) are more likely to enter HE than students from non-academic households (i.e., neither parent has completed HE; [2]). In Germany, for example, 86% of students from academic households, compared to 76% of students from non-academic households, enroll in HE after graduating from upper secondary school [3].

Importantly, it constitutes both an individual and a societal loss that students from non-academic households are discouraged from entering HE, despite being qualified. On the individual level, achieving a HE diploma is arguably the most promising opportunity for social upward mobility [4]. On the societal level, HE graduates are an increasingly precious resource for the success of a countries’ economy [5]. Failing to identify and support talented students that actually have the potential to successfully attain HE is thus troublesome.

Corresponding to this significance, increasing social equality in students’ HE enrollment is a topic of high political interest, and governments invest great amounts of money to reduce social disparities. For example, Germany spent 12.9 billion Euros on the support of (disadvantaged) individuals in the education system in 2017 [6].

Likewise, the topic of social equality in HE enrollment has also received much scientific attention in the last two decades. Not only the success of political programs are frequently evaluated [6], various disciplines closely monitor the influence of societal and economic changes to gain a deeper understanding of social disparities in HE enrollment and its underlying mechanisms [7, 8]. A recent incident that has arguably brought massive changes to individuals and societies is the outbreak of COVID-19 and the subsequent pandemic.

In the present research, we examine students’ intention to enter HE in 2021, the second year of the COVID-19 pandemic in Germany. By means of a theoretically elaborated model, we aim to provide valuable insights into the main sociological and psychological sources responsible for social disparities and where existing patterns of inequalities might have shifted in the course of the pandemic.

For this, Germany provides an excellent test case as policies on school closures to the pandemic were stricter compared to most other European countries or the U.S. [9]. This may have had particularly noticeable effects on the lives of citizens in general and students in particular. Moreover, the German school system is highly stratified, leading to considerable social inequalities at different educational transitions. After completing primary school, students enter one of several lower secondary school tracks, depending primarily on their performance. Reaching upper secondary school, students constitute a relatively homogeneous, high-performing group, and performance differences between students are relatively small [10, 11].

After graduating from upper secondary school, the vast majority of students in Germany decide between two options for further educational career (around 5% of students opt for other educational pathways [12, 13]): HE or technical/vocational education and training (TVET). Pursuing HE at traditional universities or universities of applied science is basically free of charge and students are offered financial support to cover costs of living (e.g., state-subsidized student loans or scholarships). Alternatively, students opt for entering TVET programs which promise an early entry into employment, a regular income, and good long-term employment prospects in the respective sector [14].

As outlined above, the decisions students make between these two options strongly depend on the educational background of their parents [10]. To explain these social inequalities, previous research differentiated between primary and secondary effects of educational background [15]. Primary effects account for social differences in students’ academic performance, whereas secondary effects represent differences in students’ decision making behavior. Across countries, the empirical value of differentiating between primary and secondary effects in predicting HE enrollment has been demonstrated repeatedly [16]. For Germany, research has shown that 51–63 percent of social differentials in students’ intention to enter HE are due to secondary effects [17, 18]. When considering actual enrollment, secondary effects even explain more than 70 percent of social inequalities [19].

Importantly, however, the relative contribution of primary and secondary effects in explaining social inequality in HE enrollment varies depending on the institutional and economic conditions under which the educational decision is made [8, 16]. Unquestionably, the COVID-19 pandemic has severely disrupted these conditions [20, 21].

Regarding primary effects, previous research consistently shows that social differentials in academic performance are rather low among upper secondary students [18, 22]. Notably, during the COVID-19 pandemic, students had to cope with extensive class cancellations and several months of home schooling, which may have affected inequalities in students’ academic performance. In fact, knowledge gaps and declines in academic performance were particularly severe among students from non-academic households [23–26]. However, it should be taken into account that most studies reporting performance declines were based on samples of younger students (i.e., primary and lower secondary school students) [23, 27]. Upper secondary school students’ performance may have been less affected by the pandemic, as older students demonstrated more learning autonomy and activity in home schooling compared to younger students [20, 28]. Therefore, social differences in upper secondary school students’ academic performance may have increased to some extent, though not greatly, during the COVID-19 pandemic.

Relatedly, in times of COVID-19, students may have generally relied less on their academic performance when making educational decisions (e.g., between pursuing HE or TVET). In fact, students attributed performance declines to the challenges of the pandemic [20, 29]. Therefore, students may have considered their grades as less indicative of their actual academic abilities. Consequently, the relation between students’ academic performance and their intention to enter HE may have been generally weaker in times of COVID-19. As a result, academic performance may have had a similar or even smaller explanatory contribution to social inequalities in students’ HE enrollment as compared to pre-COVID-19 cohorts.

Regarding secondary effects, *rational choice theory* serves as a well-established theoretical framework to describe and examine secondary effects in students’ educational choices [30, 31]. Accordingly, for each option (i.e., HE and TVET) students consider expected *benefits* (e.g., employment prospects), expected *costs* (e.g., direct costs), as well as their *subjective probability of success*. We outline research on social inequalities in these three dimensions, their impact on students’ intention to enter HE, and potential influences of the COVID-19 pandemic on these relations in what follows.

In pre-COVID-19 cohorts, social differences in students’ evaluations of benefits of HE were found to be rather negligible [10, 18]. During the pandemic, however, social inequalities in expected benefits of HE may have increased. Specifically, contact restrictions led students to reduce their exchanges with people outside their own families and close social circle. Additionally, student counseling services, career information events, and university open days were largely canceled. This may have limited upper secondary school students in their exploration of career opportunities outside of their family environment [28, 32]. Consequently, students from non-academic households may have been biased against pursuing HE and, for instance, underestimated a university degree’s benefits on the labor market. By contrast, students from academic households probably received family members’ first-hand information about HE and, thus, evaluated a university degree’s benefits more favorably. Therefore, social differences in students’ evaluation of employment prospects associated with HE may have increased in times of COVID-19, and, in turn, contribute to social inequalities in students’ intention to enter HE [10, 18].

Whereas past research merely focused on the utility value (i.e., employment prospects) when capturing students’ evaluations of benefits of HE [10, 18], we additionally capture the attainment value attached to HE (i.e., students’ personal significance of pursuing HE) [31, 33]. Specifically, students from non-academic households may have attributed less personal significance to pursuing HE in times of COVID-19, lacking academic role models and reference to university life. In contrast, most students from academic households may have developed a sense of personal significance of pursuing HE, aspiring to follow their parents’ steps [33]. Therefore, social differences in students’ personal significance of pursuing HE may be considerably large during the pandemic. We furthermore expect that HE may attract especially those who perceive it as personally significant, since today’s young people value a feeling of belonging and fulfillment in career options [34, 35]. Consequently, students’ personal significance of pursuing HE may also contribute to social inequalities in their intention to enter HE.

Students’ evaluation of costs associated with HE was highly related to their educational background in pre-COVID-19 cohorts. Although HE in Germany is largely tuition free, students often face high living costs in the city of their university. These costs, on average, weigh more heavily on students from non-academic households compared to students from academic households [10, 36, 37]. During the pandemic, student = ts from non-academic households may have expected it to be even more challenging to afford the costs of HE, as non-academic households on average faced heavier economic burdens due to the COVID-19 pandemic compared to academic households [38, 39]. One explanation for this is that non-academics are more likely to be employed in industries and positions that were affected by downsizing and reduced working hours due to the COVID-19 pandemic [38]. As a result, social differences in expected direct costs of HE may have been larger in times of COVID-19 compared to pre-COVID-19 cohorts. Given that students’ evaluation of costs is related to their plans for HE, expected costs may contribute to social inequalities in students’ intention to enter HE to a significant extent [10, 18].

Extending past literature, we additionally consider opportunity costs, that is the time invested in obtaining a university degree, instead of taking on a paid job. Opportunity costs are particularly burdensome for students from economically disadvantaged households [40, 41]. During the pandemic, students from non-academic households may have felt an even stronger urge to enter paid employment to avoid financial hardship for their families. Therefore, social differences in opportunity costs may have been considerably large in times of COVID-19. On the other hand, many companies faced revenue slumps and uncertain future prospects and, therefore, were temporarily reluctant to hire for apprenticeships [42, 43]. Consequently, an immediate entry into the labor market may have appeared difficult for upper secondary school graduates across social backgrounds. Therefore, expected opportunity costs may have been less predictive of students’ actual HE enrolment during the pandemic. In summary, the relation between opportunity costs and students’ intention to enter HE may be weak, even if social differences in expected opportunity costs of HE may have been considerably large in times of COVID-19.

Finally, students’ perceived probability of success in HE was highly related to their educational background in pre-COVID-19 cohorts [10, 18, 44]. During the pandemic, students lacked external information about the demands of pursuing HE, as well as about coping strategies and support services. Therefore, students from non-academic households may have struggled to evaluate their prospects to master the challenges of HE. Furthermore, given that students from non-academic households received relatively less support by their parents in home schooling than their peers from academic households [45], the former may have perceived the situation as more challenging and, consequently, also expected pursuing HE to be more demanding. For those reasons, social differences regarding students’ evaluations of their probabilities of success in HE may have been larger in times of COVID-19 compared to pre-COVID-19 cohorts. Previous studies have shown that students’ perceived probability of success in HE is highly related to their intention to enter HE [10, 18]. Consequently, students’ perceived probability of success in a large part may explain social differences in students’ intention to enter HE.

In sum, in the present research, we examine explanatory contributions of primary and secondary effects to social inequalities in the COVID-19 cohort in Germany (i.e., students attending upper secondary school during the COVID-19 pandemic). Moreover, we elaborate on social differences in students’ decision making (i.e., secondary effects) based on rational choice theory, predicting students’ intention to enter HE. Specifically, we extend previous research in capturing students’ personal significance of pursuing HE in addition to monetary benefits of HE, and opportunity costs of HE in addition to direct costs.

## Method

Study design, sample size, and exclusion criteria were preregistered (https://aspredicted.org/blind.php?x=Y5L_HJ7; note that the hypotheses outlined in the preregistration focus on a different set of research questions on variables that are not pertinent for the present manuscript). A detailed description of sample characteristics, sources of participant recruiting, and instruments of measurement can be found in the supplementary materials on the Open Science Framework (OSF, https://osf.io/dcgfa/?view_only=1bc182fcc04644b6a137c1d91077c4b0).

### Procedure

Ethics approval for the study was granted by the DIPF | Leibniz Institute for Research and Information in Education ethics committee (DIPF_EK_2021_10). Data was collected online from March 30^th^ to May 7^th^, 2021. Participants were recruited via schools, student councils, NGOs, and social media. Participants had to be at least 16 years of age and currently enrolled at upper secondary school (graduating or pre-graduating class) in Germany to be eligible for data collection. Participation took approximately 15 minutes. As incentive, fifteen gift vouchers (at 25€ each) were raffled after data collection.

We obtained written informed consent from participants, closely following the instructions of the ethics committee and the universities’ data protection officers (i.e., based on the General Data Protection Regulation of the EU). After that, participants indicated their demographics, including their age, grade, school type, and state of school. Then, intention to enter HE/TVET, subjective probability of success in HE/TVET, expected benefits, and costs of HE/TVET were assessed in randomized order (both the order of scales and items within scales were randomized). Lastly, participants were asked about their parents’ level of education, before they were thanked and debriefed. In addition, several other variables were assessed which are not pertinent for the present manuscript and will not be detailed here (see materials on OSF for more information).

### Sample

An a priori power analysis with G*Power 3.1 [46] for a linear multiple regression (fixed model, single regression coefficient) suggested a minimum of 528 participants for a two-tailed test with small effect (*f*^*2*^ = .02), alpha at .05, power at .90. Due to a large number of students interested in participating, we exceeded the pre-registered sample size. However, no analyses were conducted prior the end of data collection.

Of 1274 students who gave their consent to participate in our study, 817 students completed all items of the main study variables (i.e., intention to enter HE, subjective probability of success, expected benefits and cost of HE, educational background).

A total of 221 participants were excluded from further analysis according to preregistered exclusion criteria. Of them, seventeen participants were not eligible for the study as they were not enrolled in upper secondary school in Germany, 42 participants did not pass both attention checks, and 162 participants were identified as extreme multivariate outliers according to the Mahalanobis Distance Minimum Covariance Determinant (MH-MCD) criterion [47]. We reran all analyses with a data set to which the MH-MCD criterion was not applied observing similar patterns of results, as well as similar effect sizes. These results are reported in the supplemental analyses on the OSF. Notably, data exclusions did not differ significantly between students from academic households (*n* = 120) and students from non-academic households (*n* = 101).

The remaining sample consisted of *N* = 596 (academic households: *n* = 348; non-academic households: *n* = 248) participants, who were on average 17.36 years old (*SD* = 0.88). The majority of participants were female (64%; *N* = 363), German citizens (90%; *N* = 536), enrolled at grammar school (93%; *N* = 554), and in pre-graduating class (54%; *N* = 322).

### Measures

For the assessment of rational choice variables, students were asked to evaluate expected benefits (i.e., employment prospects and personal significance), costs (i.e., direct costs and opportunity costs), and their probability of success for both HE and TVET on 6-point Likert scales ranging from 1 = *completely disagree* to 6 = *completely agree*. In line with previous literature (calculating and analyzing relative benefits of HE over TVET; [18]), we then took the difference of both ratings, with positive values indicating a higher rating for HE (and negative values indicating a higher rating for TVET). This also applies to students’ intention to enter HE. That is, we calculated the differences between students’ intention to enter HE and students’ intention to enter TVET. Again, positive values indicate a higher rating for HE (and negative values indicating a higher rating for TVET).

#### Educational background

Students were asked about their father’s and mother’s level of education. More than 90% of participants responded to the respective single choice item. 36 open-ended responses were coded. Educational background was defined as academic (vs. non-academic) if at least one parent holds a degree in HE.

#### Academic performance

Students’ academic performance was quantified on the basis of a self-reported grade point average as well as self-reported grades in three obligatory majors (i.e., math, German, and a foreign language). Grades in upper secondary school range from 0 to 15, whereby 0 is the lowest score and 15 is the highest score. Grades from the three majors were moderately correlated (.35 < *r* < .55) and a McDonald’s omega of *ω* = .72 indicates an acceptable internal consistency. The average of major grades was in turn highly correlated with the grade point average (*r* = .86). Moreover, patterns of the mediation model (see below) did not differ between using the grade point average and the average of major grades. We therefore considered it justified to use students’ self-reported grade point average.

#### Decision making behavior

Employment prospects of HE (*ω* = .85) and employment prospects of TVET (*ω* = .88) were measured with three items each (e.g., “With a degree in HE/TVET, I would have good prospects for a well-paid job.”). Perceived personal significance of HE (*ω* = .85) and perceived personal significance of TVET (*ω* = .85) were assessed with three items each (e.g., “Pursuing HE/TVET would be an enrichment in my life.”).

Direct costs of HE (*ω* = .90) and direct costs of TVET (*ω* = .87) were measured with three items each (e.g., “I would have a hard time covering the cost of pursuing HE/TVET.”). Opportunity costs of HE (*ω* = .76) and opportunity costs of TVET (*ω* = .76) were assessed with three items each (e.g., “I would waste valuable time pursuing HE/TVET.”).

Subjective probability of success in HE (*ω* = .85) and subjective probability of success in TVET (*ω* = .80) were assessed with three items each (e.g., “I would be able to master all demands to successfully graduate from HE/TVET.”).

Confirmatory factor analysis was used to test the measurement model of students’ decision making behavior encompassing five factors (i.e., employment prospects, personal significance, direct costs, opportunity costs, probability of success). The model demonstrated adequate fit (RMSEA = .043, SRMR = .041, CFI = .979, χ2(80) = 166.6, *p* < .001) [48, 49]. In addition, we tested a constraint three-factor model representing the classic rational choice model (i.e., benefits, costs, probabilities of success). However, the five-factor model was superior to the three-factor model, that is differentiating between different facets of expected benefits and costs of HE, and described the data better than not differentiating between them (χ2(7) = 1321, *p* < .001).

#### Intention to enter higher education

Intention to enter HE and intention to enter TVET were measured with one item each (“I intend to enroll in HE/TVET after graduating from upper secondary school.”). HE was described to participants as a tertiary education pathway that includes instruction at a university or universities of applied sciences. TVET was described as a postsecondary, non-tertiary education pathway that includes training in a company and/or at a technical or vocational school.

#### Control variables

We assessed students’ age, gender, nationality, mother tongue, state of school, type of school, and grade level (graduating class vs. pre-graduating class).

## Results

Means, standard deviations, and Pearson correlation coefficients for all dependent variables are shown in Table 1.

**Table 1:** Means, standard deviations, and correlations with confidence intervals of all dependent variables.

Variable	*M*	*SD*	1	2	3	4	5	6
1. Intention to enter HE	2.73	2.41						
2. Employment prospects	1.42	1.35	.41**					
			[.34, .47]					
3. Personal significance	1.18	1.60	.69**	.61**				
			[.64, .73]	[.56, .66]				
4. Direct costs	0.93	1.15	-.14**	-.10*	-.06			
			[-.22, -.06]	[-.18, -.02]	[-.14, .02]			
5. Opportunity costs	-0.41	1.38	-.57**	-.45**	-.66**	.17**		
			[-.62, -.51]	[-.51, -.38]	[-.70, -.61]	[.09, .24]		
6. Probability of success	-0.64	0.83	.40**	.07	.28**	-.23**	-.35**	
			[.34, .47]	[-.01, .15]	[.21, .36]	[-.31, -.15]	[-.42, -.28]	
7. Grade point average	11.05	1.78	.38**	.19**	.33**	-.00	-.31**	.33**
			[.31, .45]	[.11, .27]	[.25, .40]	[-.08, .08]	[-.38, -.23]	[.25, .40]

*M* and *SD* are used to represent mean and standard deviation, respectively. Values in square brackets indicate the 95% confidence interval for each correlation.

* *p* < .05.

** *p* < .01.

We examined the relationship between students’ educational background (academic vs. non-academic households) and their intention to enroll in higher education (HE), and to what extend the relation is mediated by primary effects (i.e., academic performance) and secondary effects (i.e., employment prospects, personal significance, direct costs, opportunity costs, and probabilities of success in HE). We controlled for age, gender, grade level, type of school, nationality, and mother tongue. Educational background was coded as non-academic = 0 and academic = 1.

**Fig 1:**
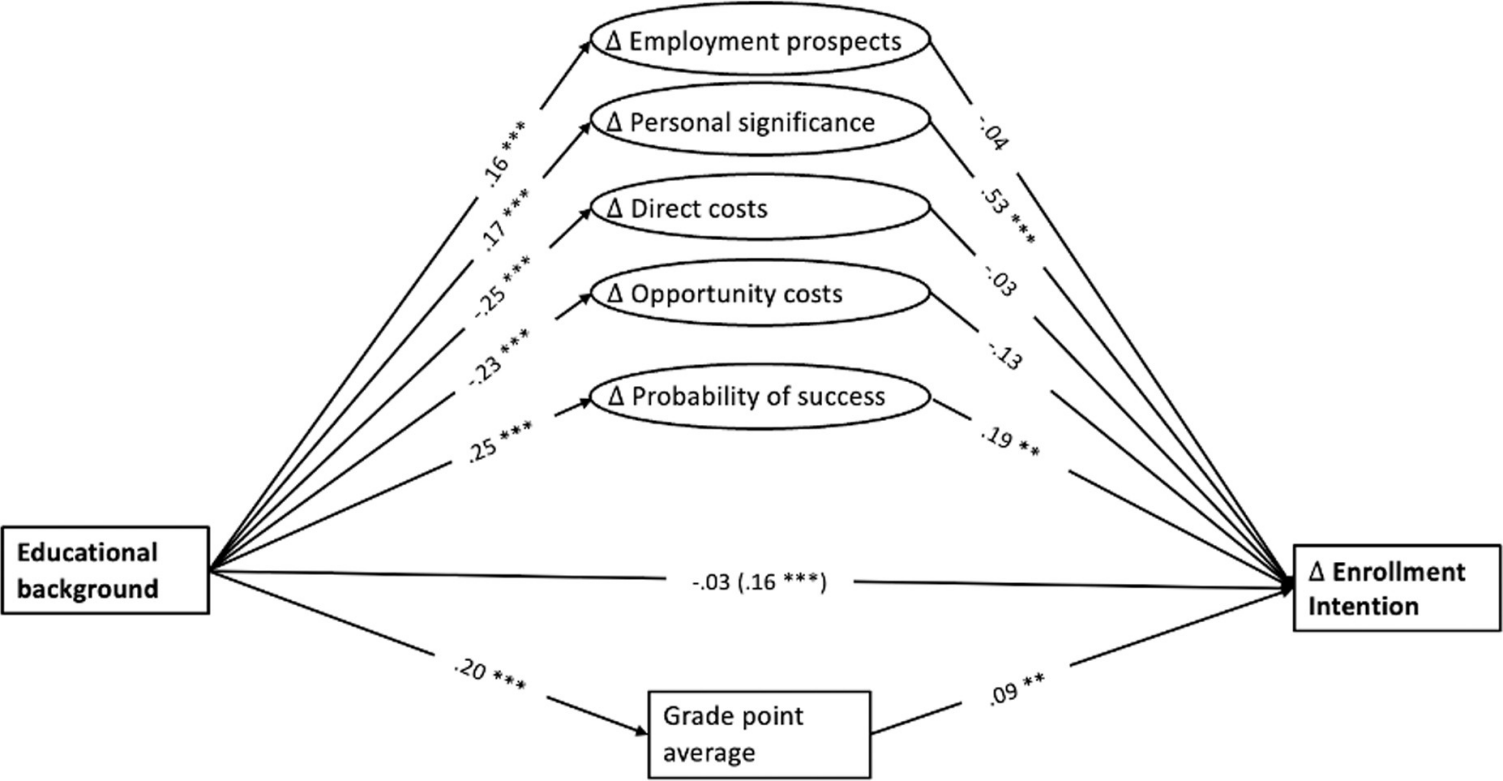
Standardized coefficients of the mediation model of educational background on intention to enter HE through rational choice patterns and academic performance. Standardized linear regression coefficients are presented. The total effect is presented in brackets. Educational background: 0 = non-academic household; 1 = academic household. We controlled for age, gender, grade level, type of school, nationality, and mother tongue. * *p* < .05. ** *p* < .01. *** *p* < .001.

Statistical analyses were conducted using *R* (Version 4.0.2), and the packages *lavaan* [50] and *oaxaca* [51]. In order to test the proposed mediation model, we estimated path analysis using latent constructs for students’ decision making behavior. Bootstrapping (*k* = 5000) was applied to estimate model fit, as well as the direct and indirect effects of the hypothesized mediation model [52]. Standardized path coefficients of this mediation model are displayed in Fig 1. Indirect effects of students’ educational background on their intention to enter HE through the discussed mediators can be found in Table 2. Furthermore, we ran a Blinder-Oaxaca Decomposition to determine the relative importance of primary and secondary effects [51]. Note that we also performed the mediation analysis with employment prospects, direct costs, probabilities of success in HE, and academic performance as mediators only, mirroring past research that did not specifically test the roles of personal significance and opportunity costs as additional mediators [18]. Detailed results of these analyses can be found in the OSF.

**Table 2:** Indirect effects from educational background on intention to enter HE through rational choice patterns and academic performance.

Indirect effects from educational background^a^ on intention to enter HE	β	*SE*	*z*	95% CI		*p*
				*LL*	*UL*	
through employment prospects	-.01	0.04	-0.71	-0.03	0.01	.475
through personal significance	.09	0.14	3.08	0.04	0.15	.002
through direct costs	.01	0.06	0.70	-0.01	0.03	.485
through opportunity costs	.03	0.09	1.50	-0.01	0.07	.133
through probability of success	.05	0.08	2.85	0.02	0.09	.004
through performance	.02	0.04	2.05	0.01	0.03	.040

β = standardized coefficient; *SE* = standard error; *z* = z-value; CI = confidence interval; *LL* = lower limit; *UL* = upper limit.

^a^ 0 = non-academic household, 1 = academic household. We controlled for age, gender, grade level, type of school, nationality and mother tongue.

### Relative explanatory contribution of primary and secondary effects in students’ intention to enroll in higher education

The full mediation model demonstrated adequate fit (RMSEA = .037, SRMR = .043, CFI = .964, χ2(206) = 368, *p* < .001) [48, 49]. Academic performance and rational choice variables explained substantial amounts of variance in students’ intention to enter HE (R^2^ = .57).

Overall, rational choice variables (i.e., secondary effects) explained 76.79% of social differences in students’ intention to enter HE (β = .17), whereas academic performance (i.e., primary effects) explained 13.95% (β = .02).

### Primary effects

On average, students from academic households reported a higher grade point average than their peers from non-academic households. Students’ educational background was significantly related to their academic performance (β = .20, *SE* = 0.15, *z* = 4.82, *p* < .001, CI 95% [0.12, 0.28]). Moreover, the higher students’ performance, the stronger was their intention to enter HE. That is, students’ academic performance was significantly related to their intention to enter HE (β = .09, *SE* = 0.05, *z* = 2.27, *p* = .023, CI 95% [0.02, 0.16]). After all, academic performance partly explained social differences in students’ intention to enter HE, as indicated by a significant indirect effect of students’ educational background on their intention through academic performance (β = .02).

### Secondary effects

Regarding expected benefits of HE, students from academic households expected higher employment prospects and perceived greater personal significance of pursuing HE than students from non-academic households. That is, students’ educational background was significantly related to expected employment prospects of HE (β = .16, *SE* = 0.11, *z* = 3.55, *p* < .001, CI 95% [0.07, 0.25]), as well as to personal significance of HE (β = .17, *SE* = 0.14, *z* = 3.71, *p* < .001, CI 95% [0.08, 0.26]). However, expected employment prospects were not related to students’ intention to enter HE (β = -.04, *SE* = 0.10, *z* = -0.76, *p* = .446, CI 95% [-0.14, 0.06]), and the indirect effect of students’ educational background on their intention through expected employment prospects was non-significant (β = -.01). By contrast, the more students perceive pursuing HE as personally significant, the stronger was their intention to enter HE. That is, students’ personal significance of HE was strongly related to their intention to enter HE (β = .53, *SE* = 0.16, *z* = 5.53, *p* < .001, CI 95% [0.36, 0.74]). Moreover, students’ personal significance of HE partly explained social differences in students’ intention to enter HE, as indicated by a significant indirect effect of students’ educational background on their intention through personal significance (β = .09).

Students from non-academic households expected higher direct costs and opportunity costs of HE than students from academic households. That is, students’ educational background was significantly negatively related to expected direct costs of HE (β = -.25, *SE* = 0.11, *z* = -5.13, *p* < .001, CI 95% [-0.35, -0.16]), as well as to expected opportunity costs of HE (β = -.23, *SE* = 0.13, *z* = -4.97, *p* < .001, CI 95% [-0.32, -0.14]). However, neither expected direct costs (β = -.03, *SE* = 0.10, *z* = -0.72, *p* = .470, CI 95% [-0.12, 0.05]) nor expected opportunity costs of HE (β = -.13, *SE* = 0.13, *z* = -1.62, *p* = .106, CI 95% [-0.27, 0.04]) were significantly related to students’ intention to enter HE. Likewise, data revealed no indirect effects of students’ educational background on their intention through direct costs (β = .01) and opportunity costs (β = .03).

Finally, students from academic households expected a higher probability of success in HE than students from non-academic households. That is, students’ educational background was significantly related to their subjective probability of success in HE (β = .25, *SE* = 0.07, *z* = 4.77, *p* < .001, CI 95% [0.15, 0.35]). Besides, the higher students’ subjective probability of success, the stronger was their intention to enter HE. That is, students’ subjective probability of success in HE was significantly related to their intention to enter HE (β = .19, *SE* = 0.21, *z* = 3.48, *p* = .001, CI 95% [0.09, 0.31]). Moreover, subjective probability of success in HE partly explained social differences in students’ intention to enter HE, as indicated by a significant indirect effect of students’ educational background on their intention through subjective probability of success (β = .05).

## Discussion

Despite being of high political and societal interest, the decision to enter HE strongly depends on the social and educational background of students’ parents. That is, upper secondary school graduates from non-academic households less often enter HE compared to their peers from academic households [3, 53]. In the present research, we investigated social inequalities in students’ intention to enter HE, as well as underlying mechanisms of rational choice (i.e., expected benefits, costs, and probabilities of success) in the second year of the COVID-19 pandemic in Germany. Extending past research, we also captured personal significance and opportunity costs of HE. Importantly, the following discussion of our results in light of the research from pre-COVID-19 cohorts (i.e., discussing potential effects of the COVID-19 pandemic) is only based on results that were robust across analyses of both the extended and the classic rational choice model.

In general, the present research yielded two main results. First, students’ academic performance explained a minor share of social inequalities in students’ intention to enter HE. In line with recently published studies, students from non-academic households reported a lower academic performance than their peers from academic households [3, 25, 26]. However, we found only weak associations for the relation between academic performance and students’ intention to enter HE, whereas data from pre-COVID-19 cohorts showed moderate associations [18, 44]. A relatively weak relation between academic performance and students’ intention to enter HE implies that students did not rely much on their grades when making educational choices in times of COVID-19. A reason for this may be that students were skeptical about whether their grades during the pandemic represent their actual academic abilities. Precisely, students may have attributed performance losses to school closures during the pandemic, and performance gains to teachers’ lower demands during home schooling [20].

Second, social inequalities in students’ intention to enter HE were largely explained by students’ decision-making behavior (i.e., secondary effects). Specifically, students from non-academic households evaluated benefits of HE (i.e., employment prospects and personal significance), costs of HE (i.e., direct costs and opportunity costs), and probabilities of success less favorably than their peers from academic households. These social differences in students’ evaluations of costs of HE and probabilities of success in HE were moderate, which is in line with research on pre-COVID-19 cohorts [10]. Interestingly, we also found moderate social differences in students’ evaluations of job market benefits of HE, whereas in data from pre-COVID-19 cohorts, social differences in expected relative employment prospects of HE were rather negligible [18]. Finally, the higher students’ personal significance of HE, and the higher students’ subjective probability of success, the stronger was students’ intention to enter HE.

### Theoretical implications

In this study, we replicated well-established theoretical models for the analysis of primary effects and secondary effects explaining social differences in students’ HE enrollment [15, 30, 31] in the context of the COVID-19 pandemic. Importantly, we extended existing literature in two ways.

First, past literature merely focused on utility values (i.e., employment prospects) when capturing students’ evaluations of benefits of HE [10, 12, 18]. In addition, we also captured attainment values attached to HE by students’ personal significance of pursuing HE [31, 33]. Indeed, personal significance was found to have a major explanatory contribution to social inequalities in students’ intention to enter HE. This is in line with recent literature arguing that today’s young people generally value personal significance (e.g., interest, enjoyment, and meaning) in career opportunities over monetary or status gains [34, 35, 54]. This may explain the strong relation between students’ personal significance of pursuing HE and their intention to enter HE.

Interestingly, employment prospects associated with HE and students’ intention to enter HE were not related in the extended rational choice model. Moreover, employment prospects did not serve as a mediator of social differences on students’ intention to enter HE in this model. This contrasts past research that found employment prospects to be weakly related to students’ intention to enter HE [18]. A possible explanation for this is that students’ evaluations of employment prospects and their personal significance of pursuing HE share considerable amounts of variance. Arguably, students may perceive pursuing HE as personally significant because of decent employment prospects associated with a university degree. As a result, students’ considerations about employment prospects would not predict their intention to enter HE above and beyond their personal significance of pursuing HE.

Second, previous research mostly focused on direct costs of HE [10, 12, 18]. Herein, we additionally measured expected opportunity costs as another dimension of costs associated with pursuing HE. Interestingly, however, expected opportunity costs and students’ intention to enter HE were not related. One may speculate whether this may be due to a temporarily limited supply of apprenticeships and job opportunities in times of COVID-19 [43]. That is, students may have expected to have fewer opportunities as alternatives to pursuing HE during the pandemic. Nonetheless, capturing opportunity costs of HE can be considered relevant in Germany, where pursuing HE is free of charge but time consuming. Furthermore, our findings of social inequalities in expected opportunity costs of HE may reflect social differences in risk perceptions. The financial and time investment of pursuing HE is associated with multiple risks, such as sudden financial hardship, course failure, and dropout [55]. Students from non-academic households are on average more risk averse regarding educational investments compared to their peers from academic households [56, 57]. This can be explained by *prospect theory* [56, 58]. Accordingly, students from non-academic households perceive an academic career as an opportunity for status gain, whereas for students from academic households pursuing HE is a necessity to avoid status loss. Thus, students from non-academic households are less willing to take risks when taking career steps than their peers from academic households [58]. Promising early entry into the labor market, technical/vocational education and training (TVET) is a low-risk alternative to HE. Therefore, TVET is considered an attractive career opportunity by many students from non-academic households, which they sacrifice when entering HE [59, 60].

Likewise, expected direct costs of HE and students’ intention to enter HE were not related. Moreover, direct costs could not serve as a mediator of social differences in students’ intention to enter HE. In Germany, where tuition is free and students have to afford living costs only, the relation between expected direct costs and students’ intention to enter HE is typically relatively small [18]. In times of COVID-19, this association may have been even smaller, given that students could have expected to continue education from home (i.e., home schooling), not forcing them to take on additional costs for living. This may explain why expected direct cost of HE and students’ intention to enter HE were non-related in our data.

### Practical implications

The present results further give insights into how social inequalities in students’ HE enrollment may be reduced. Performance deficits of students from non-academic households (i.e., primary effects) partly explained social inequalities in students’ intention to enter HE. The present data further suggests that these deficits may be larger in the second year of the pandemic compared to pre-COVID cohorts [18]. To counteract this, students from non-academic households may benefit from additional learning support to catch up with their peers from academic households, a strategy that has already been employed by NGOs and schools [61].

However, tutoring is not enough, as students’ decision making behavior (i.e., secondary effects) explained a relatively larger share of social inequalities in students’ intention to enter HE. Consequently, information- and resource-based interventions that reduce differences in expected benefits, costs, and probabilities of success in pursuing HE may promise to be more fruitful [62, 63].

#### Expected benefits of higher education

Compared to past research, social inequalities in expected relative employment prospects seem to be larger in the second year of the COVID-19 pandemic. This may be due to a stronger parental influence in times of COVID-19. During the pandemic, students spent most of their time in their family environment and received comparatively little information about HE from external sources. Lacking external information about a university degree’s benefits, students from non-academic households may have expected worse employment prospects with a university degree than their peers from academic households. Therefore, informing students about employment prospects associated with a university degree may be effective in reducing social inequalities in students’ evaluations of benefits of HE [18, 40, 62].

More importantly, students from non-academic households may benefit from directly experiencing university life to develop a sense of personal significance of pursuing HE. Indeed, our findings imply that students’ expected personal significance is the strongest predictor of students’ intention of all rational choice variables observed. Prior to the pandemic, high school students had the chance to visit universities or to get in touch with university students (e.g., university open days). These possibilities were largely reduced during the COVID-19 pandemic, for example, because university open days were postponed without replacement. However, our results yielded evidence for the value of such visitor events, in particular to reduce social inequalities. Based on our findings, we suggest that those events may focus on sparking a sense of personal significance of pursuing HE in students.

#### Expected costs of higher education

The provision of financial support for students was expanded extensively during the COVID-19 pandemic (e.g., interim financial aid; [64]). Yet, in our data, students from non-academic households expected direct costs of HE to be more burdensome compared to their peers from academic households. Indeed, most students from non-academic households lack information about scholarships and student grants in Germany [65]. This points to the necessity of facilitating access to scholarships and financial aid, as well as encouraging students more actively to apply for it [40].

#### Subjective probability of success in higher education

Likewise, social differences in students’ subjective probability of success may be explained by a lack of information about the demands of HE, and how to master them. Therefore, students from non-academic households may benefit from comprehensible and easily accessible information about how to cope with daily challenges in HE. For instance, university graduates from non-academic households could share their experiences in pursuing HE, and thereby act as role models for upper secondary students [66].

In addition to information provision, hands-on support is essential to increase the subjective probability of success in HE of students from non-academic households [67]. Providing practical help may be particularly important in times of COVID-19, as students from non-academic households, on average, could not rely as much on their parents’ support in home schooling compared to their peers from academic households [45]. For instance, schools and universities may provide (technical) support in the enrollment process, in virtual classes, and in everyday university life. Moreover, mentoring was found to be effective in supporting disadvantaged students [68].

### Limitations and future research

Before concluding, several limitations ought to be discussed. First, our cross-sectional design does neither allow for causal inferences on the links within our model (however, see for causal evidence: [18, 40, 62]), nor on how the pandemic impacts social disparities in students’ intention to enter higher education and underlying mechanisms. We merely have the chance to highlight differences between our results and studies on pre-COVID-19 cohorts based on narrative comparison.

Second, we collected data from a convenience sample, which generally poses the risk of self-selection bias. Therefore, our sample may suffer from an over-representation of respondents with certain characteristics (e.g., a specific social background). For instance, most participants of our study attended grammar schools (i.e., German Gymnasium). Since access to grammar schools is more selective than access to other types of upper secondary schools in Germany, grammar school students form a relatively homogeneous group. In fact, they are on average higher performing and more socio-economically privileged, compared to students attending comprehensive schools or vocationally oriented schools [69]. Remarkably, we nevertheless find notable social differences in our sample, and social inequalities may be even larger when including a greater variety of upper secondary school types.

Moreover, our study only focused on HE and TVET, while neglecting other potential pathways of the German postsecondary educational system. For instance, in some programs (e.g., “duales Studium”) students attend university as part of their employment at a company. Those students may perceive costs and benefits of HE differently than students pursuing regular study programs and, thus, our results may not easily apply to such paths [12]. However, the vast majority (i.e., approximately 95%) of upper secondary school graduates in Germany opt for either HE or TVET [12].

Third, we assessed students’ intention to enter HE instead of measuring students’ actual enrollment decisions. Importantly, students’ intention to enter HE is strongly associated with their actual enrollment [70]. This applies in particular to pre-graduating students, a majority of whom have already made a decision about their academic career prior to their final year [13, 71]. Future research may analyze actual enrollments at universities in the upcoming months to validate our findings (e.g., the DZHW Panel Study of School Leavers with a Higher Education Entrance Qualification in 2022 [72]).

Even though data collection was limited to German schools, our results may also be discussed in relation to other European countries. Although speculative, we would expect comparable patterns, at least in countries in which secondary education is similarly stratified and the transition to HE follows similar processes (e.g., the Netherlands; [73, 74]).

## Conclusion

We examined students’ intention to enter HE, as well as underlying mechanisms, in 2021, the second year of the COVID-19 pandemic in Germany. We found that social differences in students’ intention to enter HE were mainly explained by students’ decision-making behavior (i.e., secondary effects) rather than academic performance (i.e., primary effects). Our results suggest that additional to the well-documented social differences in expected costs of HE, and subjective probability of success in HE [18]. social differences in expected benefits of HE might be larger in times of COVID-19 as compared to pre-COVID-19 cohorts. In sum, our findings point to the need for interventions to address rational choice patterns of students from non-academic households (i.e., evaluations of benefits, costs, and probabilities of success), counteracting social disparities in students’ educational choices.
